# Cytokine release syndrome mimicking drug reaction with eosinophilia and systemic syndrome (DRESS) in a patient on blinatumomab therapy

**DOI:** 10.1016/j.jdcr.2026.06.022

**Published:** 2026-06-17

**Authors:** Sakshi Chopra, Ysaac Zegeye, Carrie Diamond, Melodi Javid Whitley

**Affiliations:** a Duke University School of Medicine, Durham, North Carolina; b Department of Dermatology, Duke University Medical Center, Durham, North Carolina

**Keywords:** cytokine release syndrome, DRESS, drug reactions, eosinophilia, medical dermatology, oncodermatology

## Introduction

Blinatumomab is a bispecific T-cell engager that treats B-cell acute lymphoblastic leukemia (B-ALL) by targeting CD19-expressing cancer cells and CD3 on effector T cells.^1^ By activating and forming a cytolytic synapse between cell types, blinatumomab triggers T-cell activation. Along with relapsed or refractory B-ALL, blinatumomab has demonstrated effectiveness in patients with minimal residual disease (MRD) positivity, helping convert patients to MRD-negative status.^2^

The potent immune activation that underlies blinatumomab’s antileukemic efficacy also contributes to one of its notable toxicities: cytokine release syndrome (CRS).^3^ CRS is a systemic inflammatory response triggered by the rapid activation of T cells upon engagement with leukemic cells.^4^ Massive cytokine release, including interleukin (IL)-1, IL-6, interferon-gamma, and tumor necrosis factor-alpha, leads to autonomic instability, cutaneous edema and rash, eosinophilia, and neurological manifestations.^4^ Rarely, CRS can present similarly to Drug Reaction with Eosinophilia Systemic Syndrome (DRESS), a severe hypersensitivity reaction associated with certain medications.^5^ Here, we describe blinatumomab-associated CRS mimicking DRESS in a patient receiving treatment for B-ALL, with rapid resolution on oral steroids.

## Case report

A 65-year-old female patient with a history of poorly controlled Type 2 diabetes mellitus and psoriasis presented with a subacute history of gastrointestinal distress, chest pain, and dyspnea. Additional workup revealed significant leukocytosis with hypoproliferative bone marrow; flow cytometry and bone marrow biopsy confirmed Philadelphia-positive B-ALL. She underwent a 28-day induction regimen with dasatinib (100 mg orally daily) and prednisone (10 mg/m^2^ orally daily), along with blinatumomab administered in 4 28-day continuous-infusion cycles (28 mcg IV daily) separated by 14-day treatment-free intervals.

Dermatology was consulted on day 18 of the first blinatumomab cycle for a 2-day history of a new, severely pruritic rash. Examination showed facial and neck edema, mild erythema, and scattered petechiae without morbilliform eruption or lymphadenopathy (Fig 1). Laboratory evaluation revealed worsening thrombocytopenia, rising eosinophilia (absolute eosinophil count increased from 0.45 to 2.24 × 10^9^ cells/L), and new-onset transaminitis (AST 124 U/L, ALT 59 U/L). She was hemodynamically stable and afebrile. With a RegiSCAR score of 2, both blinatumomab-associated CRS and DRESS were considered. Although eosinophilia is more typical of DRESS, CRS can rarely present with eosinophilic predominance due to IL-5 overproduction during lymphoblast expansion. Despite diagnostic uncertainty at this stage between a diagnosis of CRS or DRESS, empiric treatment with systemic corticosteroids was initiated because either disease would likely respond to this treatment.

**Fig 1:**
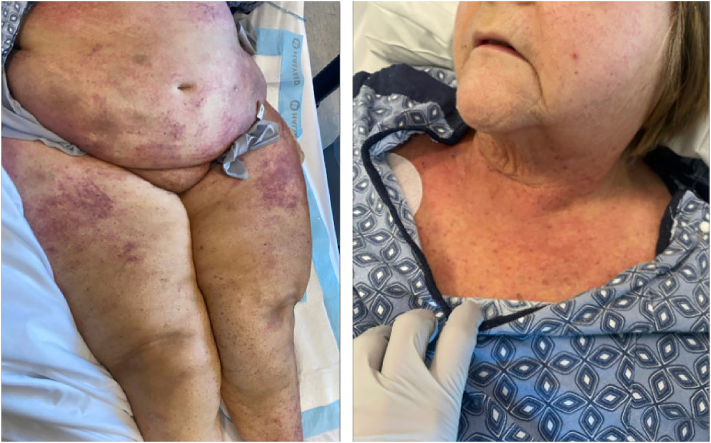
Physical examination on day 18 of the first cycle of blinatumomab. Left: Diffuse morbilliform eruption involving the trunk and lower extremities. Right: Marked facial and cervical erythema with associated edema.

The patient was started on oral prednisone 60 mg daily, chosen over intravenous therapy due to poorly controlled diabetes and concern for candiduria. Eosinophilia resolved within 48 hours, and prednisone was discontinued after 3 days as cutaneous and systemic symptoms improved. After discontinuation of prednisone, the patient did not experience any recurrence of any symptoms, including pruritus, transaminitis, or eosinophilia. Her swift response to such a short course of steroid therapy, along with her capacity to tolerate the original chemotherapeutic medication through the rest of the treatment cycle, allowed for the likely diagnosis of CRS in this patient, given that patients with DRESS typically require long steroid courses to prevent relapse.^6^ Blinatumomab therapy continued without interruption, and she remained without recrudescence for an additional 40 days until discharge.

## Discussion

CRS is a rare, potentially life-threatening condition characterized by a rapid effector cell-mediated cytokine response to immunotherapy.^4^ IL-1, IL-6, and interferon-gamma are active in the pathogenesis of fever and systemic inflammation in CRS.^4^ Early research has demonstrated symptom reversal with tocilizumab, an anti-IL-6 receptor antibody, and corticosteroids.^7^ However, given the potential of abrogating the anti-cancer effects of immunotherapy, treatment is not universally beneficial.

Multiple B-ALL therapies, including blinatumomab and chimeric antigen receptor (CAR) T-cell therapy, are associated with CRS.^3^ However, recognition and effective management of CRS while maintaining antileukemic treatment remain challenging. Clinical recognition is challenging due to heterogeneous presentations and overlap with syndromes like DRESS, including rash and edema, and management remains individualized to balance toxicity control with preservation of antileukemic activity.

This case highlights an atypical presentation of CRS with edema and peripheral eosinophilia mimicking DRESS, a clinically similar but pathophysiologically distinct entity (Fig 2). CRS responded rapidly to oral corticosteroids, suggesting that high-dose intravenous steroids or prolonged immunosuppression may not always be required. Importantly, discontinuation of blinatumomab was unnecessary, underscoring the need to balance CRS management with continuation of effective antileukemic therapy.

**Fig 2:**
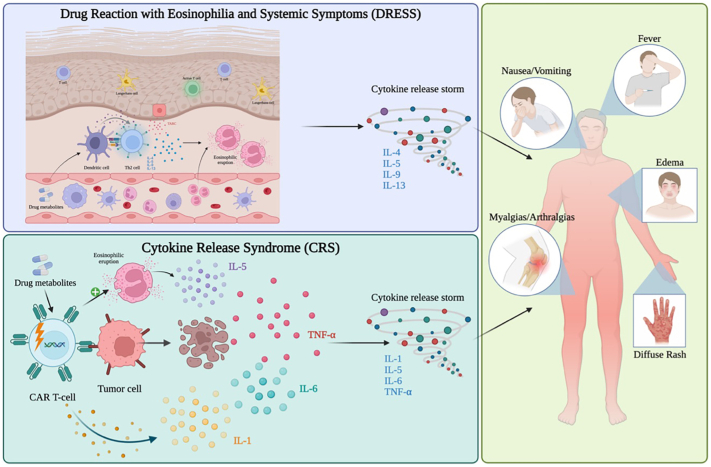
Pathogenesis and clinical presentation of DRESS, an eosinophilic drug reaction, and CRS, an inflammatory response of T cells following activation via immunotherapy.

## Conflicts of interest

None disclosed.
